# Role of pulmonary hemodynamics in determining 6-minute walk test result in atrial septal defect: an observational study

**DOI:** 10.1186/s13019-018-0725-6

**Published:** 2018-05-22

**Authors:** Supomo Supomo, Handy Darmawan, Adika Zhulhi Arjana

**Affiliations:** 1 Department of Thoracic and Cardiovascular Surgery, Dr. Sardjito General Hospital, Faculty of Medicine, Public Health and Nursing, Universitas Gadjah Mada, Kesehatan St. Number 1, Sleman, Yogyakarta, 55281 Indonesia; 2 Faculty of Medicine, Universitas Islam Indonesia, Yogyakarta, Indonesia

**Keywords:** Atrial septal defect, Cardiac catheterization, Hemodynamic, Pulmonary hypertension

## Abstract

**Background:**

The presence of altered pulmonary hemodynamics in adult patients with atrial septal defect (ASD) is common. However, there are no observational studies which evaluate the impact of altered pulmonary hemodynamics on the 6-min walk test (6MWT) result. This study aimed to investigate the role of pulmonary hemodynamics in determining 6MWT result of patients with ASD.

**Method:**

Forty-six consecutive adult patients with ASD were included in this study. Right heart catheterization was performed to obtain the pulmonary hemodynamics profile. Meanwhile, 6MWT was presented as high or low with cut-off point 350 m. Receiver operating characteristic (ROC) was used for analytical methods.

**Result:**

Abnormal functional capacity was indicated by ROC result of mPAP cut-off value of > 24 mmHg (*p =* 0.0243; AUC = 0.681). The value of PVR > 3.42 woods unit (WU) showed high specificity in determining abnormal functional capacity (*p* = 0.0069; AUC = 0.713). Flow ratio with cut-off point ≤4.89 had the highest sensitivity (100%) (*p =* 0.8300; AUC = 0.520).

**Conclusion:**

Pulmonary hemodynamics can serve as an indicator of 6MWT result in adult ASD patients with values of mPAP> 24 mmHg and PVR > 3.42 WU.

## Background

Atrial septal defect (ASD) is a common congenital heart disease (CHD) with 1.64 per 1000 living birth prevalence and female predominance [1]. Ninety percent of the patients were reported to survive into adulthood and 35% of them developed secondary pulmonary hypertension (PH) [2, 3], which is defined as increased mean pulmonary artery pressure (mPAP) ≥25 mmHg in right heart catheterization (RHC) [4].

The presence of altered pulmonary hemodynamics due to secondary PH in adult patients with ASD is associated with reduced survival and high hospital utilization [5, 6]. Previous studies had presented the impact of altered pulmonary hemodynamics due to primary PH to functional capacity of the patient, which was measured by the 6-min walk test (6MWT) [7, 8]. The 6MWT result represents the functional capacity and predicts the outcome of the patient with PH. Values of 6MWT distance less than 350 m are a predictor of worse outcome [9].

In our knowledge, there has not been any observational studies which evaluate the impact of altered pulmonary hemodynamics due to secondary PH on adult patients with ASD on the 6MWT result. We conducted this study to investigate the role of each of the components of pulmonary hemodynamics in determining the 6MWT result.

## Methods

### Study participants and ethical consideration

Between January 2014 and March 2017, 46 consecutive patients with ASD in Dr. Sardjito General Hospital were included in this study. The inclusion criteria were: adults above 18 years of age, who were diagnosed with ASD. The diagnosis of ASD was confirmed by either transthoracic echocardiography or transesophageal echocardiography. This study included subjects who had not underwent ASD closure or vasodilator therapy to decrease PAP. This study was approved by the Institutional Ethics Committee of Faculty of Medicine, Public Health and Nursing of Universitas Gadjah Mada, Indonesia and the need for individual informed consent was waived.

### Study protocols and definitions

All of the subjects underwent RHC to obtain their pulmonary hemodynamic profile. Pulmonary hemodynamic profile components analyzed in this study were mPAP, pulmonary vascular resistance (PVR), and flow ratio. Flow ratio is defined as ratio of Qp and Qs taken from RHC data.

$$ {Q}_p=\frac{{\mathrm{O}}_2\ \mathrm{consumption}\ \left(\mathrm{mL}/\min \right)}{\mathrm{PV}\ {\mathrm{O}}_2\ \mathrm{content}\ \left(\mathrm{mL}/\mathrm{L}\right)\hbox{-} \mathrm{PA}\ {\mathrm{O}}_2\ \mathrm{content}\ \left(\mathrm{mL}/\mathrm{L}\right)} $$

$$ {\mathrm{Q}}_{\mathrm{s}}=\frac{{\mathrm{O}}_2\ \mathrm{consumption}\ \left(\mathrm{mL}/\min \right)}{\mathrm{SA}\ {\mathrm{O}}_2\ \mathrm{content}\ \left(\mathrm{mL}/\mathrm{L}\right)\hbox{-} \mathrm{PA}\ {\mathrm{O}}_2\ \mathrm{content}\ \left(\mathrm{mL}/\mathrm{L}\right)} $$

PV (pulmonary vein); PA (pulmonary artery); SAO2 (blood oxygen saturation); MVO2 (myocardial volume oxygen).

High flow ratio is defined as Qp/Qs > 2.36 [10]. The PH was diagnosed when mPAP was ≥25 mmHg in RHC. The 6MWT was performed after RHC to obtain the information regarding the functional capacity of the patients according to American Thoracic Society guidelines [11]. It was performed indoors on a long, flat, straight, and hard surface. This test measures the distance in meters which a patient can quickly reach unassisted in 6 min. The abnormal functional capacity was defined as 6MWT distance less than 350 m. All patients were not provided supplemental oxygen while undergoing the 6MWT. Echocardiography data was used as a baseline data for further ASD evaluation.

### Statistical analysis

**Fig. 1:**
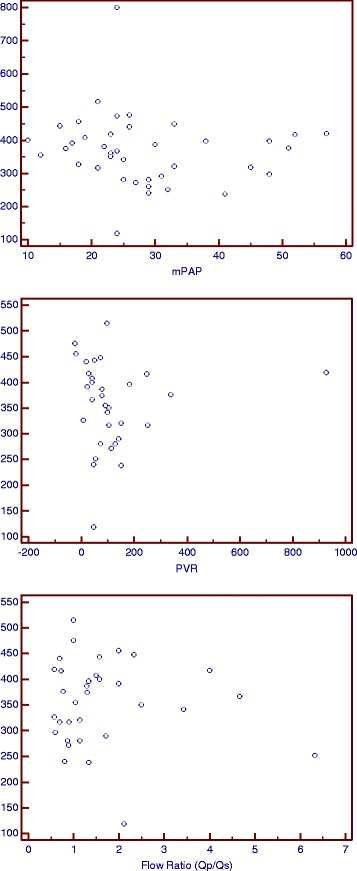
mPAP, PVR, and Flow Ratio correlating to 6MWT plot analyzed with Spearman’s rank correlation test (*r* = − 0.329 *p* = 0.0238; *r* = − 0.339 *p* = 0.0212; *r* = − 0.002 *p* = 0.9850)

**Fig. 2:**
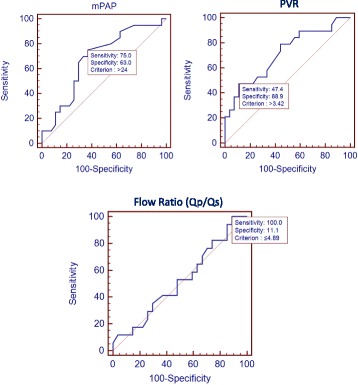
Result of ROC analysis of mPAP, PVR, and Flow Ratio in determining abnormal functional capacity (6MWT < 350 m)

Baseline characteristics of the patients are shown in Table 1. Continuous variables were presented as mean ± standard deviation (SD) and categorical variable was presented as percentage. Continuous variables with non-parametric data were presented in median. On bivariate analysis, difference between PH and non-PH is presented on Table 2. The results are considered to be significant if *p* <  0.05. The relationship between PH and functional capacity was analyzed using chi-square test (Table 3). In addition, correlations between each pulmonary hemodynamics component and 6MWT were analyzed using Spearman’s rank correlation test due to their non-parametric data (Fig. 1). The ROC analysis was used for analyzing capability of mPAP, PVR, and flow ratio in determining functional capacity of the patient, which was presented as binary data of normal or abnormal (Fig. 2). Statistical review of the study was performed by a biomedical statistician. The data were analyzed using Medcalc software.

**Table 1:** Baseline characteristic of the patients

Variables	N (%)	Mean ± SD	Median (min-max)
PH	25 (54.3)		
Non-PH	21 (45.7)		
Systolic Pressure (mmHg)			110 (84–150)
Diastolic Pressure (mmHg)			70 (60–104)
Age (years)^a^			33 (18–58)
mPAP (mmHg)		28.91 ± 11.41	
PVR (WU)^a^			1.90 (0.20–16.90)
Flow Ratio^a^			2.93 (1.27–9.00)
6MWT (m)^a^			366 (119–800)
Oxygen Saturation (%)			98 (88–99)
Normal 6MWT	20 (43.5)		
Abnormal 6MWT	26 (56.5)		

^a^Non-parametric data

**Table 2:** Difference characteristic between PH and non-PH

Variables	PH	Non-PH	*p*
ASD diameter (mm)	2.84 ± 0.82	2.47 ± 0.78	0.1154
LA dimension (mm)	34.52 ± 5.3	31.86 ± 5.03	0.0727
RA dimension (mm)^a^	48 (38–69)	45 (30–50)	0.0081
LV dimension (mm)	37.45 ± 5.37	34.55 ± 4.91	0.0495
RV dimension (mm)	46.3 ± 7.8	42.41 ± 4.15	0.0384
mPAP (mmHg)	36.42 ± 9.69	19.42 ± 4.19	< 0.0001
PVR (WU)^a^	3.05 (0.99–16.90)	1.05 (0.20–3.42)	< 0.0001
Flow Ratio^a^	2.70 (1.27–4.89)	3.12 (1.50–9.00)	0.3157
6MWT (m)^a^	326 (200–462)	391 (119–800)	0.0119
High Flow	332.67 ± 73.76	357.87 ± 83.41	0.2288^b^
Low Flow	373.5 (118.2–800)^a^	357 ± 25.06	

^a^Non-parametric data; ^b^Analyzed by Kruskal Wallis

**Table 3:** Relationship between PH and functional capacity of the patient

Variables	Abnormal functional capacity	Normal functional capacity	*p*
	(6MWT < 350 m)	(6MWT ≥350 m)	
PH	15	10	0.0302
Non-PH	5	16

## Results

Twenty-five (54.3%) patients had PH with mean mPAP 28.91 ± 11.41 mmHg (Table 1). On bivariate analysis, there were significant differences of 6MWT distance between PH and non-PH patients with *p* = 0.0119 (Table 2). Mann Whitney analysis on 6MWT for high flow ratio and low ratio group showed a significant result (*p* = 0.048). Kruskal Wallis analysis for all flow ratio group showed no significant result. Relationship between PH and abnormal functional capacity of the patient was significant in chi-square analysis with *p* = 0.0302 (Table 3). Figure 1 shows the correlations between pulmonary hemodynamics and 6MWT distance. Increased mPAP and pulmonary vascular resistance (PVR) were significantly correlated with decline in 6MWT distance (*p* = 0.0238 and 0.0212, respectively). However, the correlation between increased flow ratio and declined 6MWT distance was not significant (*p =* 0.9850).

ROC analysis results are shown in Fig. 2. The mPAP cut-off value of > 24 mmHg had 63% of specificity and 75% of sensitivity in determining abnormal functional capacity (*p =* 0.0243; AUC = 0.681). The value of PVR > 3.42 woods unit (WU) was shown to have 88.9% of specificity and 47.4% of sensitivity in determining abnormal functional capacity (*p* = 0.0069; AUC = 0.713). Flow ratio had the highest sensitivity (100%) in determining abnormal functional capacity with cut-off point ≤4.89. However, this result was not significant (*p =* 0.8300; AUC = 0.520) and had a very low specificity (11.11%).

## Discussion

In adult patients with ASD, the presence of PH was reported to have association with high mortality and hospital utilization [5, 6]. The presence of PH itself is determined using the value of mPAP, which is one of the pulmonary hemodynamic components. The value of mPAP≥25 mmHg is defined as PH [4]. However, whether this abnormal value also correlates with functional limitation of the patient or not has previously not been studied. This study strengthens the link between altered pulmonary hemodynamics and functional capacity in adult patients with ASD.

This study used 6MWT as an indicator of functional capacity. Earlier studies showed that 6MWT could substitute cardiopulmonary exercise testing to measure functional capacity in low resources setting [12–14]. Several studies demonstrated the impact of altered pulmonary hemodynamics on functional capacity of the patient, which was determined using 6MWT [7, 8]. Minai et al. [7] found that patients with PH had significantly lower 6MWT compared to non-PH patients and mPAP was the best predictor of declined 6MWT distance in multivariate analysis. On the other hand, Miyamoto et al. [8] found that 6MWT significantly correlated with PVR, but was not significantly correlated with mPAP. These studies were conducted in patients with unexplained or primary PH.

In the case of altered pulmonary hemodynamics in secondary PH due to ASD, our study showed significant difference in 6MWT distance between PH and non-PH patients (Table 2). After 6MWT distance was divided into normal and abnormal functional capacity, chi-square analysis also revealed a significant relationship between those variables (Table 3). Both increased mPAP and PVR were significantly correlated with declined 6MWT distance (Fig. 1). These results demonstrated that altered pulmonary hemodynamics have an impact on functional capacity of the adult patients with ASD.

Significant difference of 6MWT results between high and low flow ratio PH patients showed that functional capacity is correlated with shunt severity. Results from ROC analysis showed flow ratio of pulmonary to systemic circulation had the highest sensitivity (100%) in determining abnormal functional capacity, but this result was not significant and had a very low specificity (11.1%).

In addition, this study also analyzed the capability of each of the pulmonary hemodynamic components in determining abnormal functional capacity of the patients using ROC analysis. Significant result was shown by mPAP and PVR. The value of mPAP> 24 mmHg (sensitivity 75%, specificity 63%) and PVR > 3.42 WU (sensitivity 47.4%, specificity 88.9%) were determined to be predictors of this abnormal condition (Fig. 2).

### Limitations

Our study has the limitation of using a cross-sectional design, which only provides the outcome and factors associated to it at a specific point of time. In addition, patients included in this study were only adult ASD patients with more than 18 years of age. Therefore, the results of this study may not be applicable for pediatric ASD patients.

## Conclusion

The presence of altered pulmonary hemodynamic on secondary PH due to ASD in adult patients is associated with abnormal functional capacity. Values of mPAP> 24 mmHg and PVR > 3.42 WU were found to be indicators of this abnormal condition.

## Abbreviations

6MWT: 6-min walk test
ASD: Atrial septal defect
CHD: Congenital heart disease
mPAP: Mean pulmonary artery pressure
PH: Pulmonary hypertension
PVR: Pulmonary vascular resistance
RHC: Right heart catheterization
SD: Standard deviation
